# Curcumin alleviates LPS-induced WI-38 cell inflammation injury by regulating PTGS2 expression

**DOI:** 10.1186/s41065-025-00441-4

**Published:** 2025-05-16

**Authors:** Hongli Xiao, Wangsheng Ma, Lin Zha, Yanmin Xiao, Hui Li

**Affiliations:** Department of Pediatrics, Puren Hospital, Wuhan University of Science and Technology, No.1 Benxi Street, Heping Avenue, Qingshan District, Wuhan City, 430081 Hubei China

**Keywords:** Infantile pneumonia, CUR, PTGS2, Molecular docking

## Abstract

**Background:**

Infantile pneumonia is a common infectious disease affecting infants and young children, which can lead to severe complications such as heart failure, significantly increasing morbidity and mortality rates among affected populations. Curcumin (CUR), a prominent natural polyphenol found in turmeric and other species of Curcuma, exhibits anti-inflammatory, antioxidant, and anticancer properties. Consequently, CUR has been hoped to be a therapeutic or preventive agent for several main human diseases. This study aims to explore the effects of CUR on lipopolysaccharide (LPS)-treated Wistsar Institute (WI)-38 cells.

**Methods:**

The cell vitality, proliferation, and apoptosis were assessed by cell counting kit-8 (CCK8) assay, 5-ethynyl-2’-deoxyuridine (EdU), and flow cytometry assays. Inflammation and oxidative stress were examined by measuring interleukins (IL)-6, IL-1β, tumor necrosis factor α (TNF-α), malondialdehyde (MDA), and superoxide dismutase (SOD) levels using the corresponding enzyme-linked immunosorbent assay (ELISA) test kits. The network pharmacology and molecule docking were carried out to predict the critical targets and potential therapeutic mechanisms of CUR in infantile pneumonia. The key target genes were predicted using PPI in the CUR protected-infantile pneumonia effect. Gene Ontology (GO) and Kyoto Encyclopedia of Genes and Genomes (KEGG) analysis were employed to exhibit the biological function. The results of prediction were confirmed in vitro experiments.

**Results:**

LPS inhibited the vitality, proliferation, and SOD levels of WI-38 cells and facilitated the cell apoptosis, IL-6, IL-1β, TNF-α, and MDA levels. CUR abolished LPS-induced regulation WI-38 cell biological functions. Besides, the 16 hub genes from potential target genes of CUR and infantile pneumonia were screened. Moreover, six hub genes (enhanced green fluorescent protein (EGFP), v-akt murine thymoma viral oncogene homolog 1 (AKT1), prostaglandin endoperoxide synthase (PTGS2), signal transducer and activator of transcription 3 (STAT3), matrix metalloproteinase 9 (MMP9), and tumor necrosis factor (TNF)) in the CUR-protected-infantile pneumonia effect were identified by PPI analysis. The therapeutic effects of CUR on infantile pneumonia might relate to anti-viral and anti-inflammatory effects predicted by GO and KEGG enrichment analysis. Interestingly, CUR repressed LPS-stimulated facilitation of PTGS2 expression. The molecular docking demonstrated that PTGS2 could directly bind to CUR. The PTGS2 levels were inhibited by CUR treatment and negatively related to the time after WI-38 cells were treated with cycloheximide (CHX). PTGS2 knockdown could promote LPS-induced injury in WI-38 cells. CUR expedited cell vitality and proliferation and suppressed cell apoptosis, inflammation, and oxidative stress in LPS-induced WI-38 cells via down-regulating PTGS2.

**Conclusion:**

CUR attenuates LPS-induced WI-38 cell injury by downregulating PTGS2. CUR may be the potential drug for alleviating LPS-induced WI-38 cell inflammation damage via regulating PTGS2 expression.

**Supplementary Information:**

The online version contains supplementary material available at 10.1186/s41065-025-00441-4.

## Introduction

Infantile pneumonia is a prevalent respiratory disease in infants and young children and is characterized by pathogen infections, such as viruses, bacteria, and fungi in the lower respiratory tract [1, 2]. At present, most treatments for pneumonia in infants and young children are antiviral and antibacterial. However, with the increased use of antibiotics, the increase in resistance of pathogenic bacteria makes the disease difficult to cure [3]. Lipopolysaccharide (LPS) can induce an inflammatory response and has been widely used to construct infantile pneumonia models in *vitro* [4]. Hence, it is of great significance to study the occurrence and development of pediatric pneumonia and find more effective targets for the treatment of pediatric pneumonia.

Curcumin (CUR), a highly pleiotropic molecule, is derived from the root of turmeric [5]. CUR regulates anti-inflammatory actions by decreasing redox status, protein kinases, inflammatory transcription factors, cytokines, and enzymes that facilitate inflammation [6, 7]. CUR hindered the breast cancer cell inflammatory cytokines C-X-C Motif Chemokine Ligand (CXCL) 1 and −2 by regulating nuclear factor kappa B subunit 1 (NF-κB) [8]. Furthermore, CUR could improve severe pneumonia caused by influenza A virus by down-regulating macrophage inflammatory factors and inhibiting NF-κB signaling in vivo and in vitro [9]. Cheng et al*.* explored that CUR alleviated lung inflammation in lipopolysaccharide-induced neonatal rat models of acute lung damage through activating the peroxisome proliferator-activated receptor gamma pathway [10]. This study aims to exhibit the role of CUR in the inflammatory injury of Wistsar Institute (WI)−38 cells.

Prostaglandin endoperoxide synthase (PTGS2), also named cyclooxygenase 2 (COX2), belongs to cyclooxygenase enzyme, which catalyzes the conversion of arachidonic acid to prostaglandins [11]. It regulated inflammation and homeostasis by synthesizing lipid mediators such as prostaglandins, and endothelial PTGS2 promoted inflammatory disease development, including arthritis and tumors, by promoting pain, fever, and angiogenesis [12–17]. PTGS2 was involved in inflammatory responses that led to liver fibrosis and chronic periodontitis [18, 19]. Amentoflavone could reduce cartilage damage and inflammation in osteoarthritis of the knee via regulating PTGS2 [20]. Zhang et al. demonstrated that ellagic acid could improve osteoarthritis by repressing production of prostaglandin E2 (PGE2) in M1 macrophages via targeting PTGS2 [21]. Consequently, this study aims to explore the potential role of PTGS2 in WI-38 cell inflammation injury.

In this research, we aim to demonstrate the molecular mechanism of CUR on inflammation damage of LPS-induced WI-38 cells and to explore the potential role of PTGS2 in infantile pneumonia. Our research will provide a novel way to find new effective therapeutics for infantile pneumonia.

## Materials and methods

### Cell culture

In this study, the human embryonic lung fibroblast cells (WI-38) were obtained from the Zeye Biotechnology Co., Ltd. (Shanghai, China) and cultured in Dulbecco’s modified Eagle medium (DMEM) (Beyotime, Shanghai, China) supplemented with the fetal bovine serum (10%, Beyotime).

### Cell treatment and transfection

The inflammatory cell models in this research were constructed after WI-38 cells were treated with LPS. In short, the LPS (10 µg/mL, Huzhen Industrial Co., Ltd., Shanghai, China) was employed to treat the WI-38 cells for 12 h, namely the LPS group.

The WI-38 cells were exposed to different doses of CUR (2.5 µM, 5 µM, and 7.5 µM) for 24 h after being treated with LPS. The CUR (7.5 µM) was used in the following experiments. The CUR in this study was obtained from the Winherb Medical Technology Co. Ltd. (Shanghai, China).

The small interfering RNA (siRNA) for PTGS2 knockdown (si-PTGS2) and plasmids for overexpression (PTGS2) were constructed by GenePharma (Shanghai, China). The si-NC and pcDNA were used for the negative controls. The transient transfections in this research were carried out for plasmids or siRNA using Lipofectamine 3000 (Thermo Fisher Scientific, Waltham, MA, USA).

The WI-38 cells in this experiment were transiently transfected with si-NC or si-PTGS2 followed by stimulation with LPS, namely the LPS + si-NC group or LPS + si-PTGS2 group, respectively. The WI-38 cells were transiently transfected with pcDNA or PTGS2 followed by co-treatment with LPS and CUR.

After WI-38 cells were stimulated with CUR (7.5 µM), the cycloheximide (CHX, 25 µg/mL, Amerjet Technology Co., Ltd., Wuhan, China) was performed to treat the cells for 0, 6, 12, or 24 h. Subsequently, western blot was employed to test PTGS2 expression.

### Cell counting kit-8 (CCK8) assay

The cell vitality in this study was analyzed using the CCK8 test kit (Sigma, St. Louis, MI, USA). The WI-38 cells under different treatment environments were cultured into 96-well plates, and the CCK8 solution (20 µL) was added into the each well and cultivated in a humidified incubator with 5% CO_2_ and 95% air at 37℃ for h. Finally, the ability of cell vitality was examined under a microplate reader (Jukang Pharmaceutical Chemical Co., Ltd., Nanjing, China).

### 5-ethynyl-2’-deoxyuridine (EdU) assay

The cell proliferation ability was tested using the 5-ethynyl-2’-deoxyuridine EdU reagent (RiboBio Inc., Guangzhou, China). After being treated with different treatments, the cells under different treatment conditions were exposed to the EdU for 2 h, and then the paraformaldehyde (Beyotime) and 5% Triton-X-100 (Beyotime) were used to fix and permeabilize the cells. The cells were dyed with 4’,6-Diamidino-2’-phenylindole (RiboBio Inc.). The proliferation was analyzed using the fluorescence microscope (Thermo Fisher Scientific).

### Flow cytometry

For the apoptosis assay, WI-38 cells under different treatment conditions were seeded into 12-well plates. The apoptosis ability was tested using the Annexin V-fluorescein isothiocyanate (V-FITC)/PI kit (Beyotime) following the guidelines of manufacturer. The Annexin V-FITC (5 µL) and PI (5 µL) were carried out to mix with the cell suspension and cultivated for 15 min at 37℃. Ultimately, the ability of apoptosis was detected using the flow cytometry system (Thermo Fisher Scientific).

### Enzyme-linked immunosorbent assay (ELISA)

The levels of inflammation factor (interleukins (IL)−6, tumor necrosis factor (TNF-α), and IL-1β) were estimated using the IL-6, TNF-α, and IL-1β ELISA test kits (Beyotime). In short, the WI-38 cells under different treatment environments were cultured into 12-well plates, the cellular supernatants were collected, and the IL-6, TNF-α, and IL-1β concentrations were analyzed according to the instructions of manufacturer under a microplate reader (Jukang Pharmaceutical Chemical Co., Ltd.).

### Detection of cell oxidative stress

In this research, the cell oxidative stress was examined using the superoxide dismutase (SOD) activity assay kit and malondialdehyde (MDA) assay kit (Zeye Biotechnology Co., Ltd.). Briefly, the supernatants of WI-38 cells under different treatment conditions were collected. Next, the test solution was contracted with the cell supernatant according to the kit guidelines. Finally, the microplate reader (Jukang Pharmaceutical Chemical Co., Ltd.) was used to examine the MDA levels and SOD activity.

### Obtaining the CUR target and the infantile pneumonia-related gene set

The potential target gene of infantile pneumonia was predicted in the GeneCard database. The infantile pneumonia target gene set was established according to the top 1000 genes. The potential target gene of CUR was predicted using Swiss Target Prediction database. The hub genes between CUR target and infantile pneumonia-related gene were represented using the Venn diagram.

### Protein–protein interaction (PPI) analysis and enrichment analysis

The Retrieval of Interacting Genes (STRING) database in this study was carried out to construct a PPI network of hub genes between CUR targets and infantile pneumonia-related genes. The PPI network in this experiment was imported into Cytoscape 3.6.0 to explore the critical subnetwork.

The hub genes between CUR target and infantile pneumonia-related gene were used for enrichment analysis (Gene Ontology (GO) and Kyoto Encyclopedia of Genes and Genomes (KEGG) analysis) via the DAVID6.8 database (https://david.ncifcrf.gov/). Finally, the data was analyzed using microSheng letter online mapping.

### Western blot

The RIPA lysis buffer (Solarbio, Beijing, China) was employed to isolate and extract the proteins of WI-38 cells under different treatment conditions. The concentrations of total proteins were examined using the BCA test kit (Abcam, Cambridge, MA, USA), and the proteins were denatured by metal water bath (Jukang Pharmaceutical Chemical Co.) for 10 min. The total proteins were subjected to sodium dodecyl sulfate–polyacrylamide gel electrophoresis (8–15%, GE Healthcare, Piscataway, NJ, USA) and shifted to polyvinylidene fluoride (PVDF) membrane (Beyotime). Next, the PVDF membranes were probed with the anti-GAPDH (ab8245, 1:10,000, Abcam) and anti-PTGS2 (ab300668, 1:1000, Abcam) for 12 h at 4℃ and then incubated with the anti-mouse (ab6728, 1:5000, Abcam) for 2 h. Finally, the protein blots were observed under the ChemiDoc™ MP Imaging System (Bio-Rad Laboratories Inc., Hercules, CA, USA).

### Reverse-transcription quantitative polymerase chain retain (qRT-PCR)

Total RNA was extracted from cells with different tratments by TRIzol reagent (Vazyme, Nanjing, China). Following that, cDNA was synthesized with HiScript III RT SuperMix (Vazyme). The qRT-PCR was determined based on the instructions of the SYBR Green qRT-PCR Kit (Vazyme). The primers sequences used for qRT-PCR were: PTGS2, forward 5’-GTTCCACCCGCAGTACAGAA-3’ and reverse 5’-AGGGCTTCAGCATAAAGCGT-3’; GAPDH, forward 5’-GTCAAGGCTGAGAACGGGAA-3’ and reverse 5’-AAATGAGCCCCAGCCTTCTC-3’.

### Molecular docking technology

In brief, the CUR and PTGS2 inhibitor celecoxib (PMID: 35,453,005) structure was downloaded from the PubChem database. The structure of PTGS2 protein was obtained from Uniprot with the ID of 5 F19. The molecular docking of CUR against PTGS2 protein was simulated by Autodock Vina software. The PyMOL system was used to test the alignment between target objects and bond energy.

### Data analysis

The GraphPad Prism 8 software (GraphPad Software, Boston, MA, USA) in this study was employed to analyze data, which were shown as mean ± standard deviation. Each experiment was repeated at least 3 times. The difference analysis was analyzed via Student’s *t*-test or analysis of variance (ANOVA) followed by Tukey’s test. The significance level was presented as * *P* < 0.05, ** P < 0.01, and *** P < 0.001.

## Results

### CUR hinders the LPS-stimulated WI-38 cell inflammation damage

**Fig. 1:**
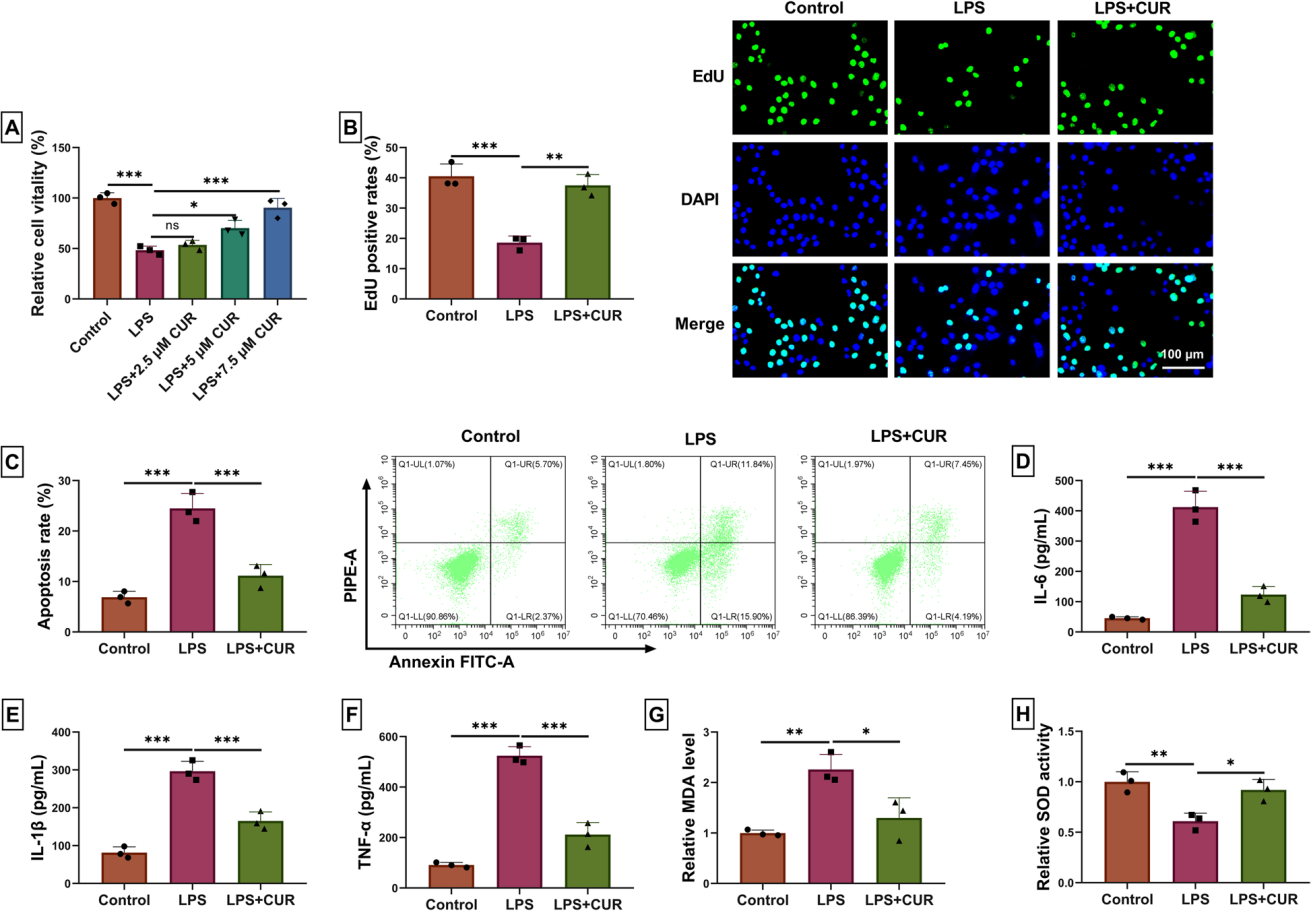
The LPS-stimulated WI-38 cell inflammation damage is inhibited by CUR. After WI-38 cells were treated with LPS, these cells were treated with different doses of CUR (2.5 µm, 5 µm, and 7.5 µm). **A** The cell vitality was detected by CCK8 assay. **B** The EdU assay was used to test the cell proliferation. **C** The ability of apoptosis was analyzed using flow cytometry. **D**-**F** The inflammation factor production was examined using corresponding kits. **G** The levels of MDA were examined using MDA assay kit. **H** The SOD activity assay kit was carried out to analyze the SOD levels. * *P* < 0.05, ** P < 0.01, and *** P < 0.001 by one-way ANOVA and Tukey’s test

To explore the role of CUR in LPS-stimulated WI-38 cells, we tested the LPS-induced WI-38 cell biological function. The cell vitality was inhibited by the LPS, but CUR could abrogate this action with the increase of CUR concentrations (Fig. 1A), and we chose the 7.5 µm CUR for the following experiments. As shown in Fig. 1B, LPS-induced inhibition of cell proliferation was weakened by CUR. The ability of apoptosis was promoted by LPS, while CUR abolished this effect (Fig. 1C). LPS could facilitate the production of inflammation factors (IL-6, IL-1β, and TNF-α), which was counteracted by CUR (Fig. 1D-F). CUR inhibited LPS-induced facilitation of MDA levels in WI-38 cells (Fig. 1G). The SOD activity was repressed in the LPS group when compared with the Control group, but CUR reversed this action (Fig. 1H). To sum up, the CUR curbs the LPS-stimulated WI-38 inflammation injury.

### *CUR can protect infantile pneumonia *via* widespread pharmacological regulation*

**Fig. 2:**
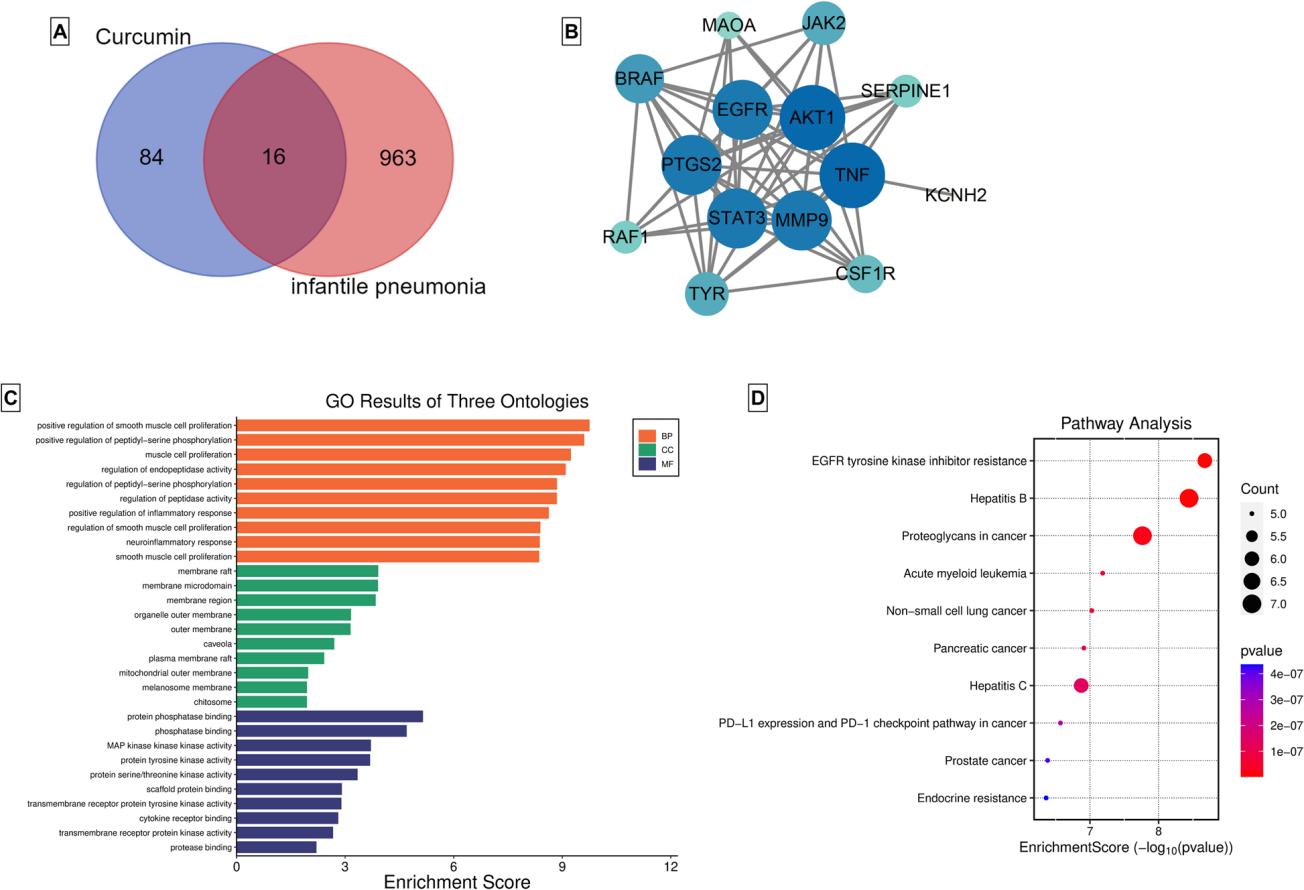
Results of network pharmacology analysis. **A** The hub genes of CUR-targeted-infantile pneumonia-related genes were shown by Venn diagram. **B** The hub genes of CUR-targeted-infantile-related genes were constructed the PPI networks using the STRING database. **C** GO analysis of hub genes. **D** KEGG enrichment analysis and pathway map of hub genes

Next, we explored whether the CUR’s protective effects on infantile pneumonia were related to pharmacological regulation. We screened 16 hub genes from potential target genes of CUR (Curcumin) and infantile pneumonia (Fig. 2A). The PPI network of hub genes showed that the genes of enhanced green fluorescent protein (EGFP), v-akt murine thymoma viral oncogene homolog 1 (AKT1), PTGS2, signal transducer and activator of transcription 3 (STAT3), matrix metalloproteinase 9 (MMP9), and tumor necrosis factor (TNF) might be the key genes of CUR targets in infantile pneumonia (Fig. 2B). As shown in Fig. 2C, the GO analysis demonstrated the positive regulation of smooth muscle and cell proliferation, positive regulation of peptidyl-serine phosphorylation, regulation of endopeptidase activity, and regulation of peptidase activity in biological processes (BP), membrane raft, membrane microdomain, organelle outer membrane, and outer membrane in cellular component (CC), and protein phosphatase binding, phosphatase binding, MAP kinase kinase kinase activity, and protein tyrosine kinase activity in molecular function (MF). The KEGG enrichment analysis showed that the hub genes had significant enrichment in EGFR tyrosine kinase inhibitor resistance, Hepatitis B, Acute myeloid leukemia, and Proteoglycans in cancer pathway (Fig. 2D). Overall, the CUR plays an important role in the progression of infantile pneumonia via the widespread pharmacological regulation.

### The CUR potential target is PTGS2

**Fig. 3:**
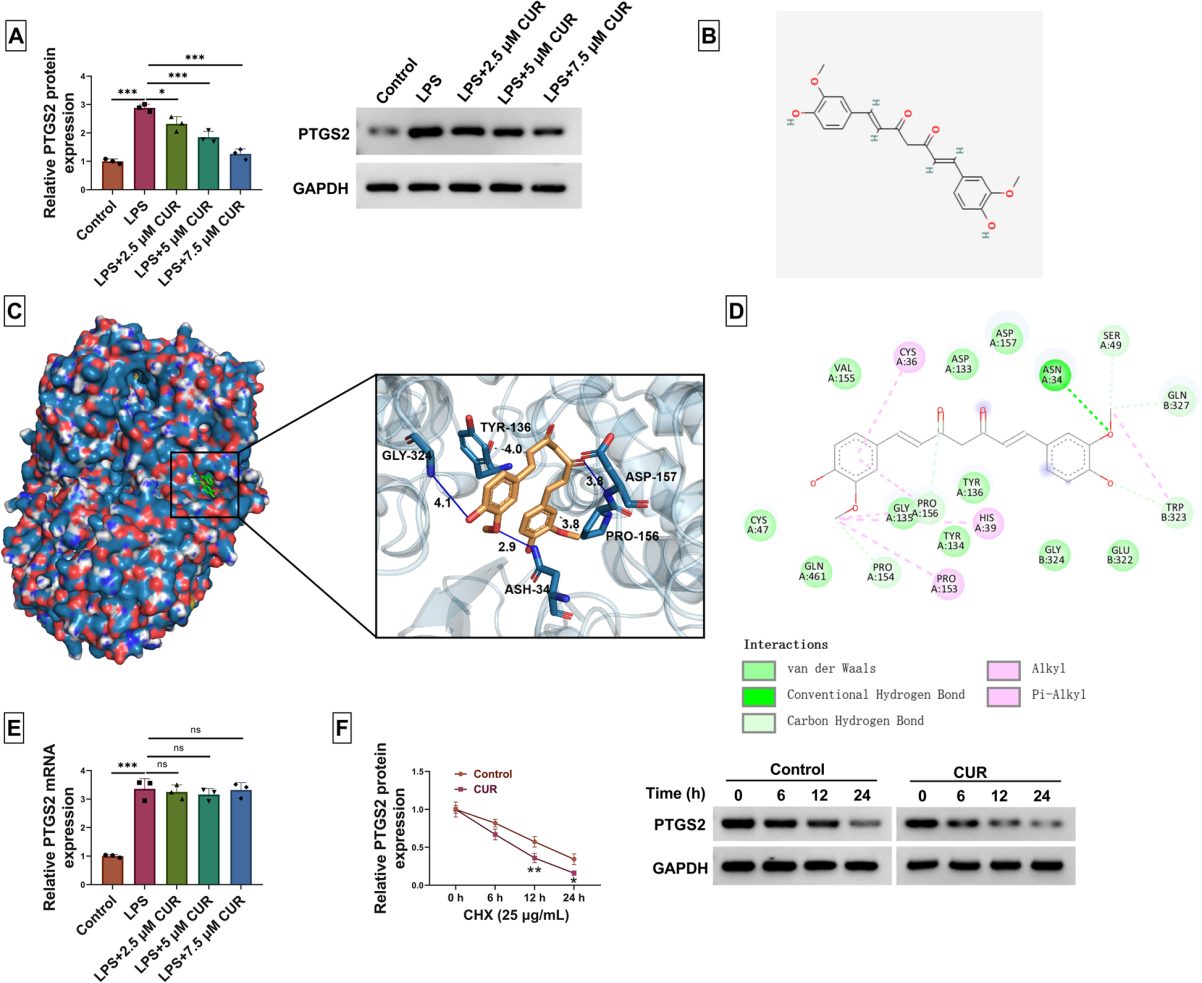
PTGS2 is a potential target of CUR. **A** The expression of PTGS2 was detected when WI-38 cells were treated with the LPS or combined with different concentrations of CUR. * *P* < 0.05 and *** P < 0.001 by one-way ANOVA and Tukey’s test. **B** The structure of CUR. **C** Biomolecular interaction of CUR and PTGS2 predicted by PyMOL system. The blue lines represent hydrogen bonds, and the yellow dash lines represent hydrophobic actions. **D** The 2D schematic diagram. On the left side, from top to bottom, were van der Waals, conventional Hydrogen Bond, and Carbon Hydrogen Bond; the right side represented Alky1 and Pi-Alky1 interactions from top to bottom. **E** The expression of PTGS2 mRNA was determined when WI-38 cells were treated with the LPS or combined with different concentrations of CUR. *** P < 0.001 by one way ANOVA and Tukey’s test, and ns means no significant difference. **F** After WI-38 cells were stimulated with CUR (7.5 µM), the CHX (25 µg/mL) was performed to treat the cells for 0, 6, 12, or 24 h. The PTGS2 protein expression was examined using western blot. * *P* < 0.05 and *** P < 0.001 by two-way ANOVA and Tukey’s test. Fig. S1: Biomolecular interaction of PTGS2 inhibitor celecoxib and PTGS2 predicted by PyMOL system

We verified the potential targets of CUR that were involved in the progression of infantile pneumonia. Due to its expression and activation during inflammation, the PTGS2/COX2-PGE2 signaling axis has been widely thought to be a driver of inflammation, induced as a direct cause of it [22, 23]. Thus, the PTGS2 was regard as the object in following experiment. As shown in Fig. 3A, LPS promoted the expression of PTGS2, while the action was weakened by CUR in a dose-dependent manner. The 2D structure of CUR was shown in Fig. 3B. The predicted docking of 3D schematic was used to show the biomolecular interaction of CUR and PTGS2, where the optimal binding energy was −7.6 kcal/mol, and CUR had 3 hydrogen bonds with GLY-324, ASH-34, and ASP-157 of the protein and hydrophobic interactions with 2 amino acid residues, TYR-136 and PRO-156 (Fig. 3C). Besides, the optimal binding energy of CUR and PTGS2 was higher than that of PTGS2 inhibitor celecoxib and PTGS2 (Fig. S1). The 2D schematic diagram was shown in Fig. 3D. Compared with the Control group, LPS promoted PTGS2 mRNA expression; CUR (2.5, 5, or 7.5 µM) did not alter PTGS2 mRNA levels when compared with the LPS group (Fig. 3E). The degradation of PTGS2 was accelerated by CUR in a time-dependent manner after WI-38 cells were treated with CHX (Fig. 3F). Hence, PTGS2 is a target of CUR.

### Knockdown of PTGS2 alleviates LPS-stimulated WI-38 cell inflammation damage

**Fig. 4:**
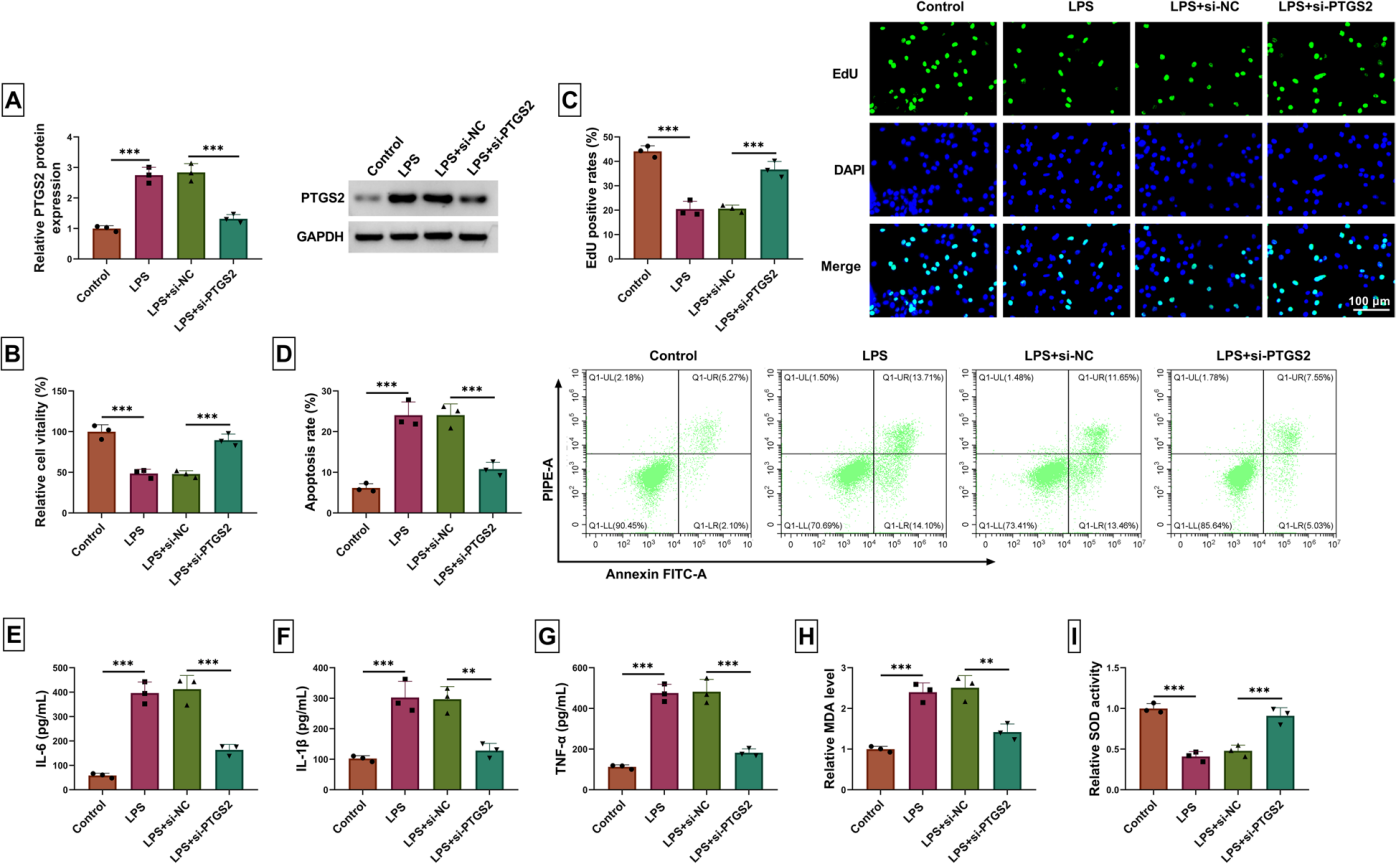
Knockdown of PTGS2 alleviates the LPS-stimulated WI-38 cell inflammation damage. WI-38 cells were transfected with si-NC or si-PTGS2 followed by treatment with 10 μg/mL LPS. **A** The PTGS2 expression was detected by western blot. **B** CCK8 was used to test the cell vitality. **C** The cell proliferation was tested by an EdU assay. **D** The ability of apoptosis was examined using flow cytometry. **E**–**G** The corresponding test kits were carried out to examine the inflammation factor productions. **H** The MDA levels were analyzed using the MDA assay kit. **I** The SOD activity assay kit was used to detect the level of SOD. ** P < 0.01 and *** P < 0.001 by one-way ANOVA and Tukey’s test

Next, the role of PTGS2 was explored in LPS-induced WI-38 cells. Downregulation of PTGS2 repressed LPS-induced facilitation of PTGS2 expression (Fig. 4A). The cell vitality of WI-38 cells after being treated with LPS was repressed compared with the Control group, while PTGS2 knockdown could rescue this effect (Fig. 4B). PTGS2 knockdown undermined the inhibitory effects of LPS on proliferation of WI-38 cell (Fig. 4C). The apoptosis was expedited after WT-38 cells were treated with LPS, while si-PTGS2 could weaken this action (Fig. 4D). The inflammation factor (IL-6, IL-1β, and TNF-α) productions were increased by LPS, which was weakened by PTGS2 knockdown (Fig. 4E-G). Downregulated PTGS2 suppressed LPS-stimulated facilitation of MDA in WT-38 cells (Fig. 4H). The LPS repressed the levels of SOD, which was reversed by si-PTGS2 (F ig. 4I). Taken together, PTGS2 knockdown hinders LPS-stimulated WI-38 inflammation injury.

### *CUR inhibits the LPS-stimulated WI-38 cell inflammation injury *via* down-regulating PTGS2*

**Fig. 5:**
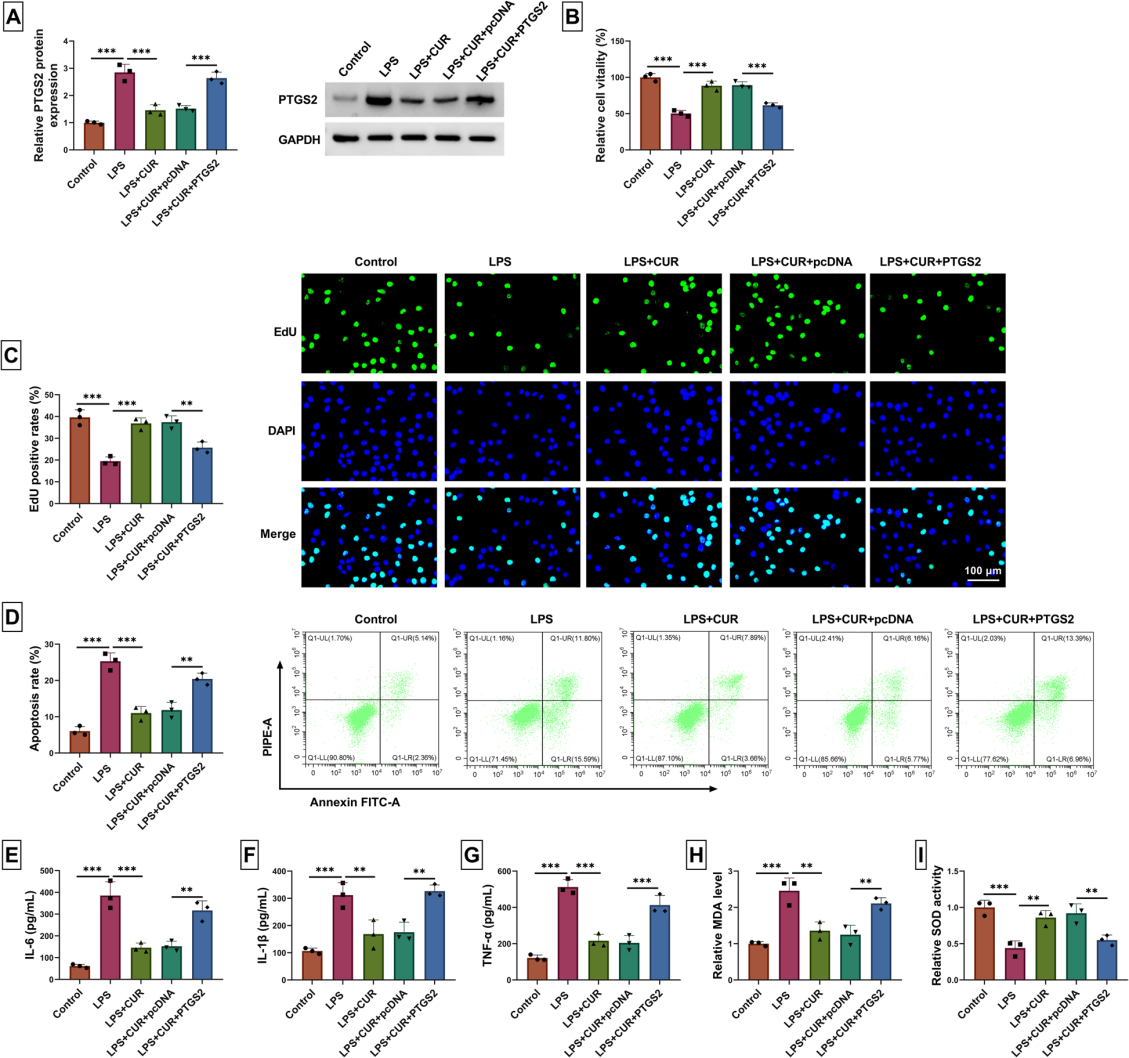
CUR inhibits the LPS-stimulated WI-38 cell inflammation injury via downregulating PTGS2. WI-38 cells were divided into five groups and named Control group (WI-38 cells), LPS group (WI-38 cells were treated with LPS), LPS + CUR group (WI-38 cells were treated with LPS and CUR), LPS + CUR + pcDNA group (WI-38 cells were transfected with pcDNA followed by treatment with LPS and CUR), and LPS + CUR + PTGS2 group (WI-38 cells were transfected with PTGS2 overexpression plasmids followed by treatment with LPS and CUR). **A** The PTGS2 levels were examined by western blot. **B** The cell vitality was analyzed using CCK8. **C** The EdU assay was used to test the cell proliferation. **D** The ability of apoptosis was examined using flow cytometry. **E**–**G** The corresponding test kits were employed to examine the inflammation factor production. **H** The MDA productions were analyzed using the MDA assay kit. I The SOD activity assay kit was carried out to detect the level of SOD. ** P < 0.01 and *** P < 0.001 by one-way ANOVA and Tukey’s test

The relationship between CUR and PTGS2 was exhibited in LPS-induced WI-38 cells. Compared with the LPS group, CUR inhibited LPS-induced promotion of PTGS2 expression, while PTGS2 overexpression could reverse this action (Fig. 5A). The cell vitality of LPS-induced WI-38 cells was expedited by CUR, which was weakened by PTGS2 up-regulation (Fig. 5B). Overexpression of PTGS2 abolished CUP-mediated facilitation of cell proliferation in LPS-induced WI-38 cells (Fig. 5C). The CUR repressed the cell apoptosis of LPS-induced WI-38 cells, but up-regulated PTGS2 could mitigate this effect (Fig. 5D). In LPS-stimulated WI-38 cells, CUR-mediated inhibition of inflammation factors was recovered by PTGS2 (Fig. 5E-G). Compared with the LPS group, CUR could retard LPS-induced promotion of MDA levels, but PTGS2 up-regulation could relieve this action (Fig. 5H). The SOD levels were promoted in LPS + CUR group when compared with the LPS group, which was abrogated by PTGS2 up-regulation (F ig. 5I). To sum up, the CUR curbs the LPS-stimulated WI-38 inflammation damage via downregulating PTGS2.

## Discussion

Pneumonia is an inflammatory disease characterized by the infection of pathogens, including viruses, bacteria, and fungi in the lower respiratory tract [24, 25]. It is a leading cause of morbidity and mortality under the age of five [26]. The primary symptoms of pneumonia in infants include shortness of breath, fever, cough, and difficulty breathing [27]. Furthermore, infantile pneumonia is one of the leading causes of mortality among children worldwide [28]. Therefore, a better understanding of how to develop active and effective therapeutic strategies for infantile pneumonia is crucial.

LPS, a gram-negative bacterial endotoxin, plays a significant role in the inflammatory response associated with infantile pneumonia [29, 30]. It has been shown that LPS induces WI-38 cell inflammation, oxidative stress, and apoptosis [31]. In this study, we explored that LPS repressed cell vitality and proliferation and increased the cell apoptosis, inflammation, MDA, and SOD levels of WI-38 cells. Our findings are consistent with those of previous studies.

CUR, a major active ingredient of Curcuma Longa, serves as both a coloring agent and a spice [32]. Additionally, it exhibits a wide range of properties, including anti-proliferative, immunomodulatory, anti-inflammatory, antioxidant, and anti-tumor effects [33–35]. CUR could reduce pro-inflammatory cytokine expression such as IL-6, IL-1, and TNF-α by preventing the degradation of iκB [36]. In mouse model experiments, the anti-inflammatory properties of CUR against lung inflammation-induced Klebsiella pneumonia had been demonstrated [37]. In our study, we found that CUR facilitated cell proliferation while inhibiting cell apoptosis, inflammation, and oxidative stress in LPS-induced WI-38 cells, aligning with previously reported experimental results.

At present, network pharmacology shows a much broader volume of data and excellent confidence due to its reliance on network theory and biological systems to topologically analyze and predict various nodes in an interconnected molecular target system [38]. Modern pharmacological studies have revealed the anti-inflammatory effects of CUR in many inflammation-related diseases [39]. Consequently, we predict the key targets and potential mechanisms of CUR in its protective effects against infantile pneumonia through network pharmacology and molecular docking, identifying 16 CUR targets associated with this condition. PPI analysis showed that EGFR, AKT1, PTGS2, STAT3, MMP9, and TNF might be key genes of CUR in infantile pneumonia. Wu et al*.* found that CUR could ameliorate heatstroke-induced lung injury by activating the PI3 K/AKT pathway [40]. Besides, in a rodent model of intestinal ischemia reperfusion, CUR played an anti-inflammatory and antioxidant role through inhibition of the NF-κB pathway in a mouse intestinal ischemia–reperfusion model of ALI [41]. GO and KEGG analysis demonstrated that the therapeutic actions of CUR on infantile pneumonia might be associated with anti-inflammatory and anti-viral roles. Thus, we suggest that in ALI, CUR may play a role in regulating the PI3 K/AKT or NF-κB signaling pathway, but further experiments are needed to confirm this.

Due to its expression and activation during inflammation, the PTGS2/COX2-PGE2 signaling axis has been widely thought to be a driver of inflammation, induced as a direct cause of it [22, 23]. Therefore, the PTGS2 is a target gene in following experiment. Notably, in LPS-induced WI-38 cells, CUR could inhibit the PTGS2 expression, and molecular docking showed that the binding energy of CUR to PTGS2 was −7.9 kcal/mol, and the binding energy of PTGS2 inhibitor celecoxib to PTGS2 was −8.5 kcal/mol, which might be associated with CUR acting through multi-target synergies and PTGS2 inhibitor celecoxib. Additionally, studies have shown that anti-inflammatory properties of curcumin stem from its ability to inhibit multiple pro-inflammatory signaling pathways mediated by NF-κB and immune cell activation. In addition, CUR modulates inflammatory mediators including cytokines, adhesion molecules, growth factors and enzymes [42]. Therefore, although CUR has a weak binding capacity with PTGS2, the overall anti-inflammatory effect may be better. We suggest that CUR may alleviate infantile pneumonia through the modulation of PTGS2.

The PTGS2 protein encoded by this gene is a potent pro-inflammatory enzyme [43]. In normal physiological conditions, PTGS2 expression is very low, and the pro-inflammatory cytokines, growth factors, and tumor promoters can directly activate the expression of PTGS2 [44]. PTGS2 knockdown reversed the facilitating effect of LPS on trophoblast invasion and inflammation in preeclampsia [45]. Additionally, Radix Paeoniae Rubra mitigated uterine inflammation in rats with pelvic inflammatory disease through si-PTGS2 [46]. In our study, we observed that LPS elevated the expression of PTGS2, while knockdown of PTGS2 suppressed the cell inflammation, apoptosis, and MDA level and promoted the cell proliferation and SOD levels in LPS-induced WI-38 cells. Moreover, CUR repressed LPS-stimulated WI-38 cell inflammation damage via PTGS2 knockdown.

Taken together, this study demonstrates that CUR alleviates LPS-stimulated WI-38 cell inflammation damage mainly by downregulating PTGS2. Our findings in this research may offer a novel direction for future clinical treatment of infantile pneumonia.

## Supplementary Information

Supplementary Material 1.

Supplementary Material 2.

## Data Availability

No datasets were generated or analysed during the current study.
