# Supplementary figures and images for: Curcumin alleviates LPS-induced WI-38 cell inflammation injury by regulating PTGS2 expression
Source: Hereditas. 2025 May 16;162:81. doi: 10.1186/s41065-025-00441-4 (PMC12083002; doi:10.1186/s41065-025-00441-4)

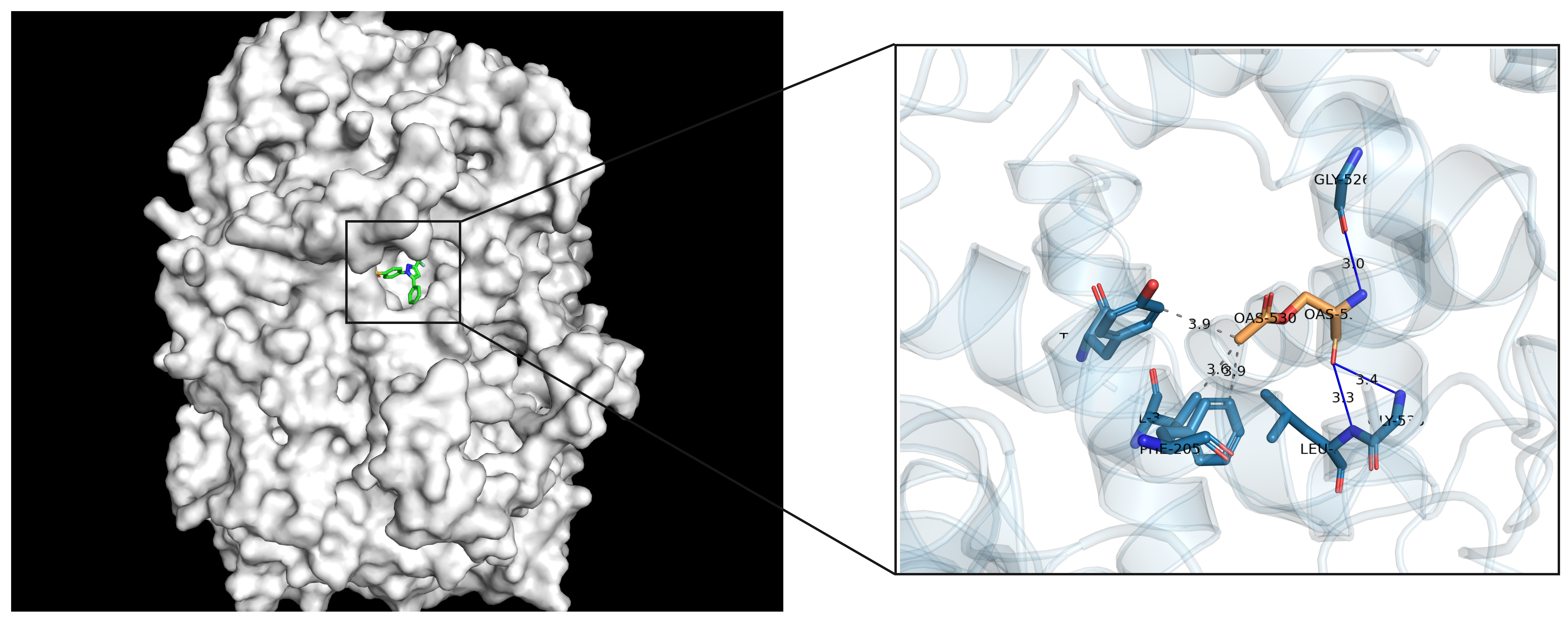

Supplement: Supplementary file 1 — Supplementary Material 1. [file 41065_2025_441_MOESM1_ESM.tif]

**Fig3 A**

**PTGS2**

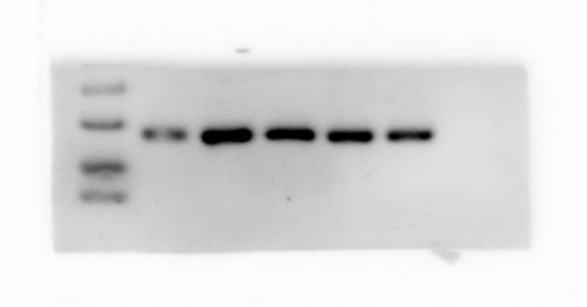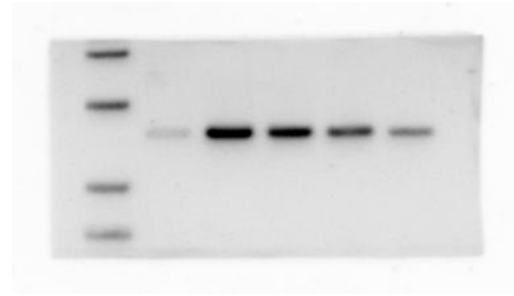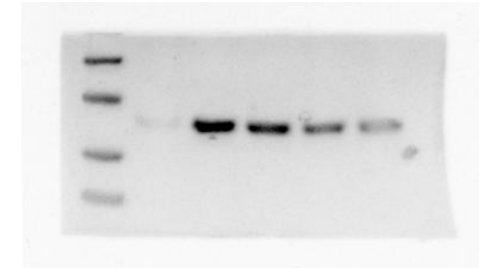

**GAPDH**

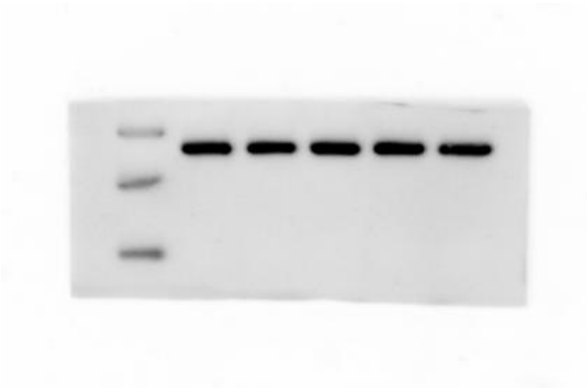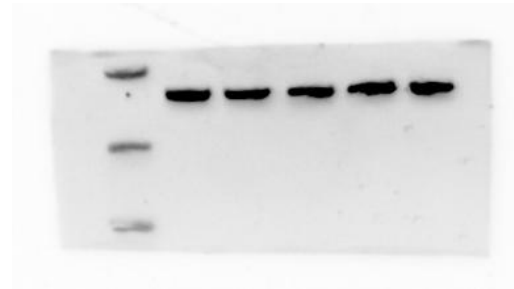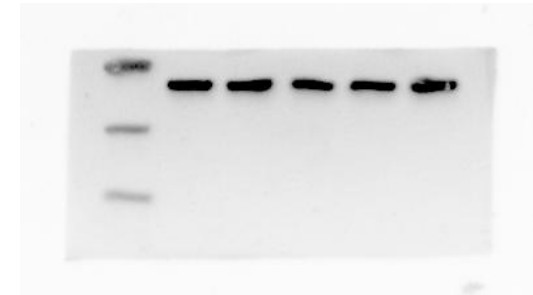

**Fig3 E**

**Control**

**PTGS2**

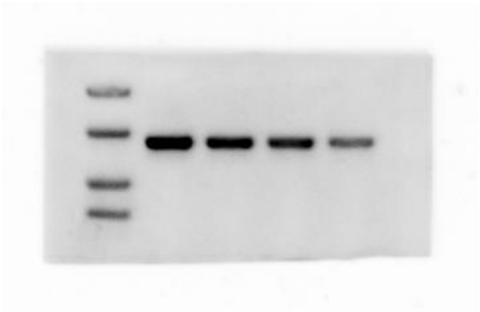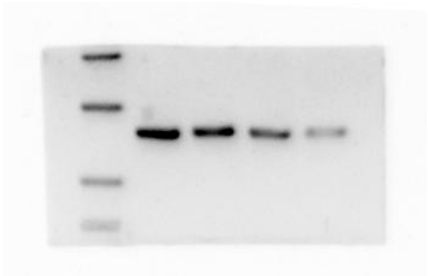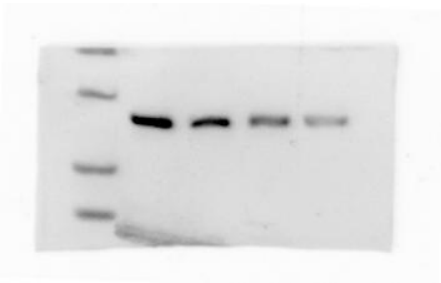

**GAPDH**

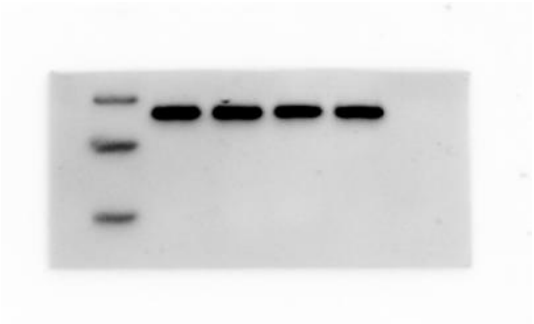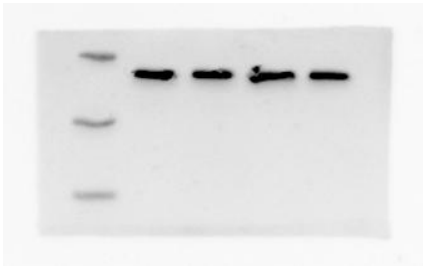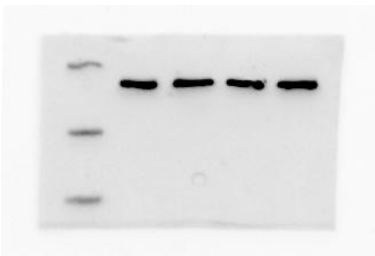

**CUR**

**PTGS2**

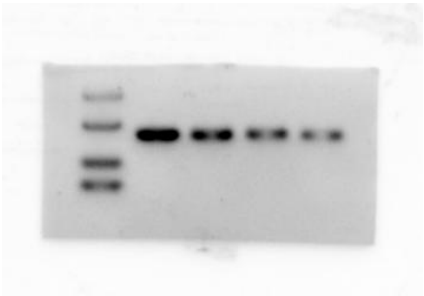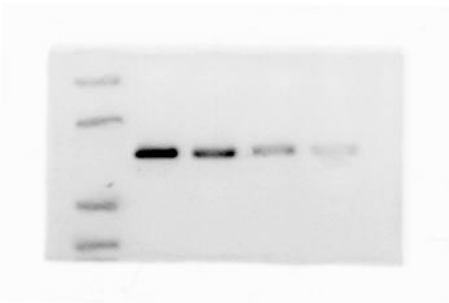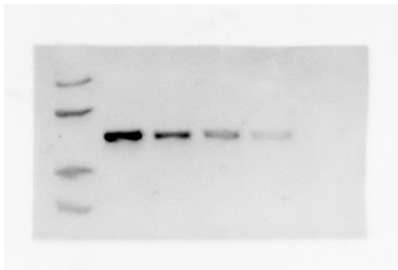

**GAPDH**

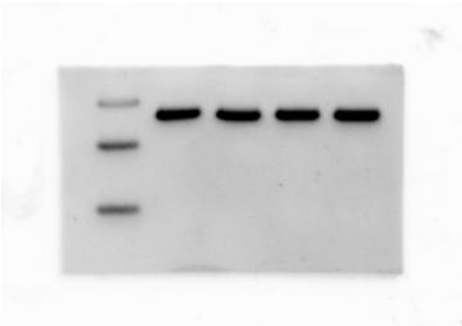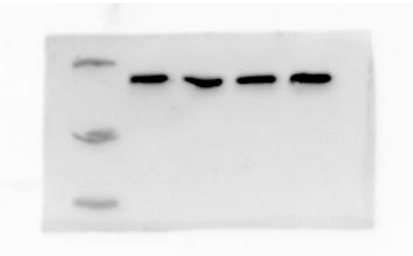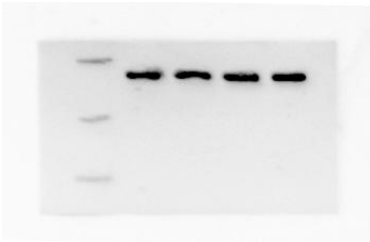

**Fig4 A**

**PTGS2**

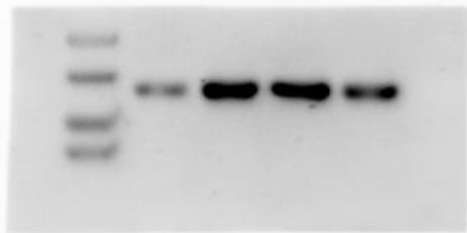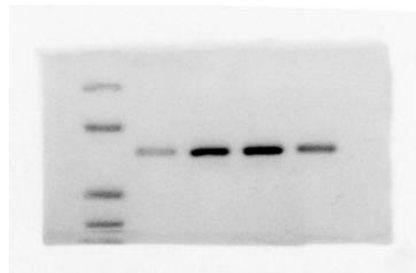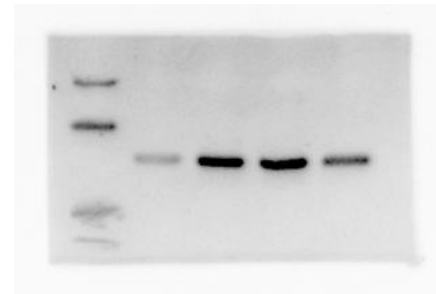

**GAPDH**

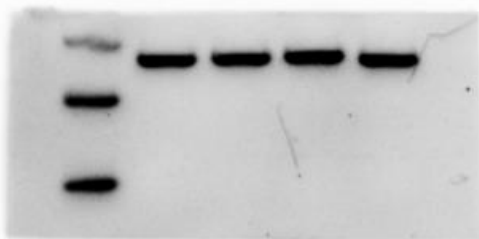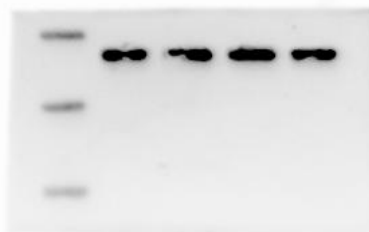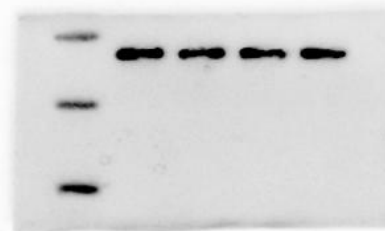

**Fig5 A**

**PTGS2**

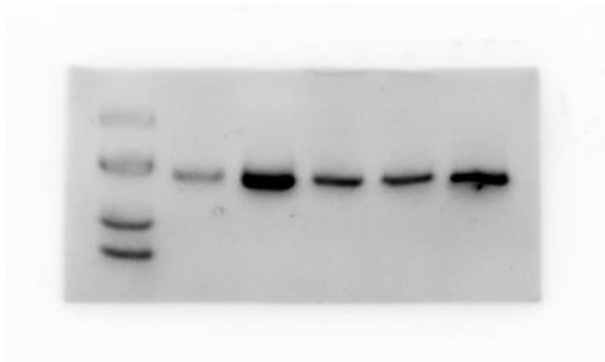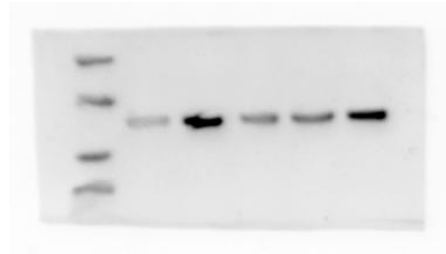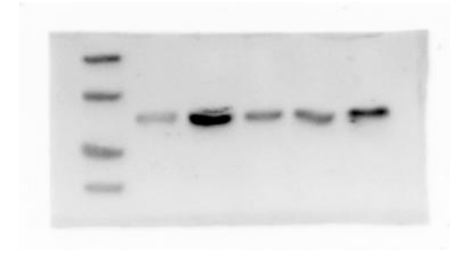

**GAPDH**

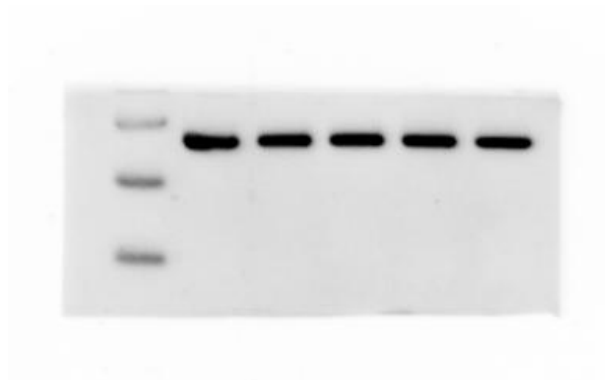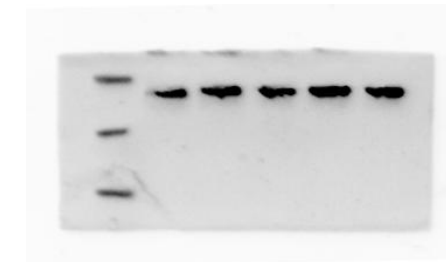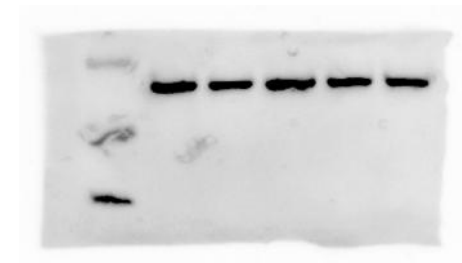

Supplement: Supplementary file 2 — Supplementary Material 2. [file 41065_2025_441_MOESM2_ESM.pdf]
